# Impact of lumbar spinal stenosis on metabolic syndrome incidence in community-dwelling adults in Aizu cohort study (LOHAS)

**DOI:** 10.1038/s41598-022-15173-y

**Published:** 2022-07-04

**Authors:** Rei Ono, Misa Takegami, Yosuke Yamamoto, Shin Yamazaki, Koji Otani, Miho Sekiguchi, Shin-Ichi Konno, Shin-Ichi Kikuchi, Shunichi Fukuhara

**Affiliations:** 1 Department of Public Health, Kobe University Graduate School of Health Sciences, Kobe, Japan; 2 Department of Physical Activity Research, National Institute of Health and Nutrition, National Institutes of Biomedical Innovation, Health and Nutrition, 1-23-1, Toyama, Shinjuku-ku, Tokyo, Japan; 3 Department of Preventive Medicine and Epidemiologic Informatics, National Cerebral and Cardiovascular Center, Suita, Japan; 4 Department of Healthcare Epidemiology, School of Public Health in the Graduate School of Medicine, Kyoto University, Kyoto, Japan; 5 Department of Environmental Epidemiology, National Institute for Environmental Studies, Tsukuba, Japan; 6 Department of Orthopedic Surgery, Fukushima Medical University School of Medicine, Fukushima, Japan; 7 Department of Community Medicine, Graduate School of Medicine, Kyoto University, Kyoto, Japan; 8 Center for Innovative Research for Communities and Clinical Excellence (CIRC2LE), Fukushima Medical University, Fukishima, Japan

**Keywords:** Epidemiology, Metabolic syndrome

## Abstract

Metabolic syndrome and lumbar spinal stenosis (LSS) are common age-related diseases. However, the causal relationship between them remains unclear. This study aimed to identify the effects of LSS on metabolic syndrome incidence in community-dwelling adults. This prospective cohort study included participants of the Aizu cohort study (LOHAS) aged < 75 years as of 2008. Participants with metabolic syndrome at baseline were excluded. The primary outcome measure was metabolic syndrome incidence, and the main explanatory variable was the presence of LSS, as assessed by a self-reported questionnaire. A multivariate Cox proportional hazard regression model was used to estimate hazard ratios (HRs) for metabolic syndrome incidence during the 6-year follow-up period. Complete-case analyses were compared with the multiple imputation results. Among 1599 participants, 1390 complete cases were analyzed (mean [SD] age 62.3 [9.0] years; females, 734 [52.8%]). Among those participants, 525 (37.8%) developed metabolic syndrome during the follow-up of 3.89 [1.96] years. The presence of LSS was associated with developing metabolic syndrome (HR, 1.41; 95% confidence interval [CI] 1.02–1.95). Multiple imputation results showed similar trends of those having complete-case data (HR, 1.47; 95% CI 1.08–2.00). This finding suggests the importance of prevention and management of LSS in community settings.

## Introduction

Metabolic syndrome, also known as syndrome X or the deadly quartet^1^, is a combination of several metabolic abnormalities. It is a major risk factor for cardiovascular diseases and a leading cause of morbidity and mortality^2^. Metabolic syndrome incidence in developed countries increases with age, occurring particularly in those older than 40 years^3,4^. Aging is also commonly associated with musculoskeletal disorders. Such an important disorder is lumbar spinal stenosis, an age-related degenerative condition in which changes occur in the discs and facet joints. The disease involves lower extremity pain, numbness, or weakness^5,6^. Studies have shown that the prevalence of lumbar spinal stenosis increases with age and is estimated to be 1.9% in respondents aged 40–49 years, 4.8% in those aged 50–59 years, 5.5% in those aged 60–69 years, and 10.8% in those aged 70–79 years^7^.

Although metabolic syndrome and lumbar spinal stenosis are common diseases whose prevalence increases with age, they are often considered separately due to their different symptoms and pathologies. Recent research has reported the coexistence of lumbar spinal stenosis and lifestyle-related diseases, such as diabetes mellitus, peripheral artery disease, hypertension, and heart disease^8–11^. However, these were not prospective studies, rather cross-sectional studies that did not identify causal relationships.

We hypothesized that lumbar spinal stenosis was a risk factor for metabolic syndrome due to extremely sedentary behavior, and the inability to meet the guidelines for physical activity^12^ could lead to metabolic syndrome. This study aimed to identify a longitudinal association between lumbar spinal stenosis and the incidence of metabolic syndrome in community-dwelling adults.

## Methods

### Study population

This prospective cohort study was based on the Locomotive Syndrome and Health Outcomes in the Aizu Cohort Study (LOHAS), with a follow-up time of up to 6 years. The LOHAS was designed in 2008 to investigate the risk of cardiovascular disease, quality of life, medical costs, and mortality attributable to locomotor dysfunction. The study recruited community-dwelling individuals from the towns of Minami-Aizu and Tadami in Fukushima Prefecture, Japan who were enrolled in a National Health Insurance plan and who participated in annual health checkups (specific health checkups targeting individuals aged < 75 years) conducted by their local governments. Both towns are in valleys surrounded by mountains, and the main industry in the region is agriculture. A detailed study protocol was provided in previous publications^13,14^. The LOHAS baseline survey included 2725 participants. The inclusion criteria for this study were having participated in the LOHAS and being aged < 75 years. The exclusion criteria were as follows: participants for whom metabolic syndrome could not be assessed at baseline owing to missing components for evaluating metabolic syndrome, those without at least one annual special health checkup until 2014, or those with metabolic syndrome at baseline. The study protocol was approved by the Research Ethics Committee of Fukushima Medical University School of Medicine and was performed in accordance with the ethical standards established in the 1964 Declaration of Helsinki and its later amendments. All participants provided written informed consent before participating in the study.

### Baseline survey

Special health checkups and questionnaires were provided at the baseline survey. All assessments were performed between April and July 2008. Each participant underwent all examinations on the same day. Special health checkups included an interview for medical history and lifestyle habits such as smoking and drinking status; measurement of weight, height, and blood pressure; physical examination; and blood chemistry tests for serum triglyceride, serum total cholesterol, serum low high-density lipoprotein cholesterol, hemoglobin A1c, and other parameters, with no instructions to fast beforehand. The baseline self-reported questionnaire for participants included information on the following characteristics: marital status, job status, dietary habits, physical activity, health-related quality of life, and locomotor dysfunction.

### Definitions of metabolic syndrome

Metabolic syndrome was defined as the presence of three or more of the following risk factors^15^, identified during the annual special or standard health checkups: (1) enlarged waist circumference (≥ 80 cm in females or ≥ 90 cm in males); (2) increased triglyceride level (≥ 150 mg/dL, or taking a drug prescribed for high triglycerides); (3) low high-density lipoprotein cholesterol (< 50 mg/dL in females or < 40 mg/dL in males, or taking drugs prescribed for low high-density lipoprotein cholesterol); (4) elevated blood pressure (systolic blood pressure ≥ 130 and/or diastolic blood pressure ≥ 85 mmHg, or receiving antihypertensive drug treatment); (5) elevated glucose (hemoglobin A1c levels ≥ 5.6% or receiving a drug prescribed for elevated glucose).

### Main explanatory variable

The main explanatory variable was lumbar spinal stenosis, which was identified using a self-reported questionnaire, developed as a diagnostic support tool for lumbar spinal stenosis^16^. This questionnaire consisted of 10 items (Q1–Q10). According to the clinical prediction rule, those with a total score of ≥ 4 points in Q1–Q4 or a score of ≥ 1 in Q1–Q4 and ≥ 2 in Q5–Q10 were identified as positive for the presence of lumbar spinal stenosis. The sensitivity and specificity of the positive criteria in a validation dataset were 84% and 78%, respectively.

### Other variables

In the special health checkup or health checkup, height and weight were measured, and the body mass index (BMI) was calculated. We recorded data on age, sex (male or female), smoking status (current or never/former), and drinking habits (every day, sometimes, rarely, or never). Data collected by questionnaires included information on employment (yes or no) and the number of metabolic syndrome components at baseline (0, 1, or 2). We also recorded the mental health domain score on the SF-36 Health Survey (higher score indicates better mental health)^17^ and the level of physical activity as assessed by the Japanese version of the International Physical Activity Questionnaire (IPAQ)^18^. The IPAQ is one of the most widely used physical activity evaluation tools in the world, and responses to the questionnaire were classified as low, moderate, or high activity, according to the scoring program^19^.

### Statistical analysis

The baseline demographics were summarized. Continuous variables are presented as mean and SD, and categorical variables as frequency and percentage. The *t*-test and chi-squared test analyzed continuous and categorical variables, respectively. The follow-up was managed as an annual special or standard health checkup. The endpoint of the present study was the development of metabolic syndrome or the last health checkup.

Cox proportional hazard regression univariable and multivariable models were used to calculate the hazard ratio (HR) and 95% confidence interval (CI) for metabolic syndrome incidence during the 6-year follow-up in association with lumbar spinal stenosis at baseline. After unavailable analysis, we performed a multivariable analysis that included age, sex, smoking status, alcohol consumption, number of metabolic syndrome components at baseline, and mental health as confounding factors. In the sub-analysis, the incidence of each of the five metabolic syndrome components and obesity (BMI ≥ 25 kg/m^2^) was analyzed in the participants by a multivariable Cox proportional hazard regression model. The outcome variables were the incidence of each of the five components or obesity during the annual special health checkups or health checkups results. The explanatory variables were the same components at baseline, lumbar spinal stenosis, and possible confounding factors (age, sex, smoking status, alcohol consumption, number of metabolic syndrome components at baseline, and mental health). A subgroup analysis stratifying participants by age (< 65 years and ≥ 65 years) was also performed.

Selection bias and loss of information due to missing data in the primary explanatory variables and covariates were managed by multiple imputations under the missing at random assumption as sensitivity analysis^20^. The variables used in the regression or logistic regression analyses were used to generate 20 imputed datasets. Rubin’s rules were applied to combine the estimates and standard errors^21^.

We also compared the complete-case analyses with the multiple imputation results. All analyses were performed using STATA, Version 17.0 (StataCorp, College Station, TX, USA). All tests were two-tailed.

## Results

### Participant characteristics

**Figure 1:**
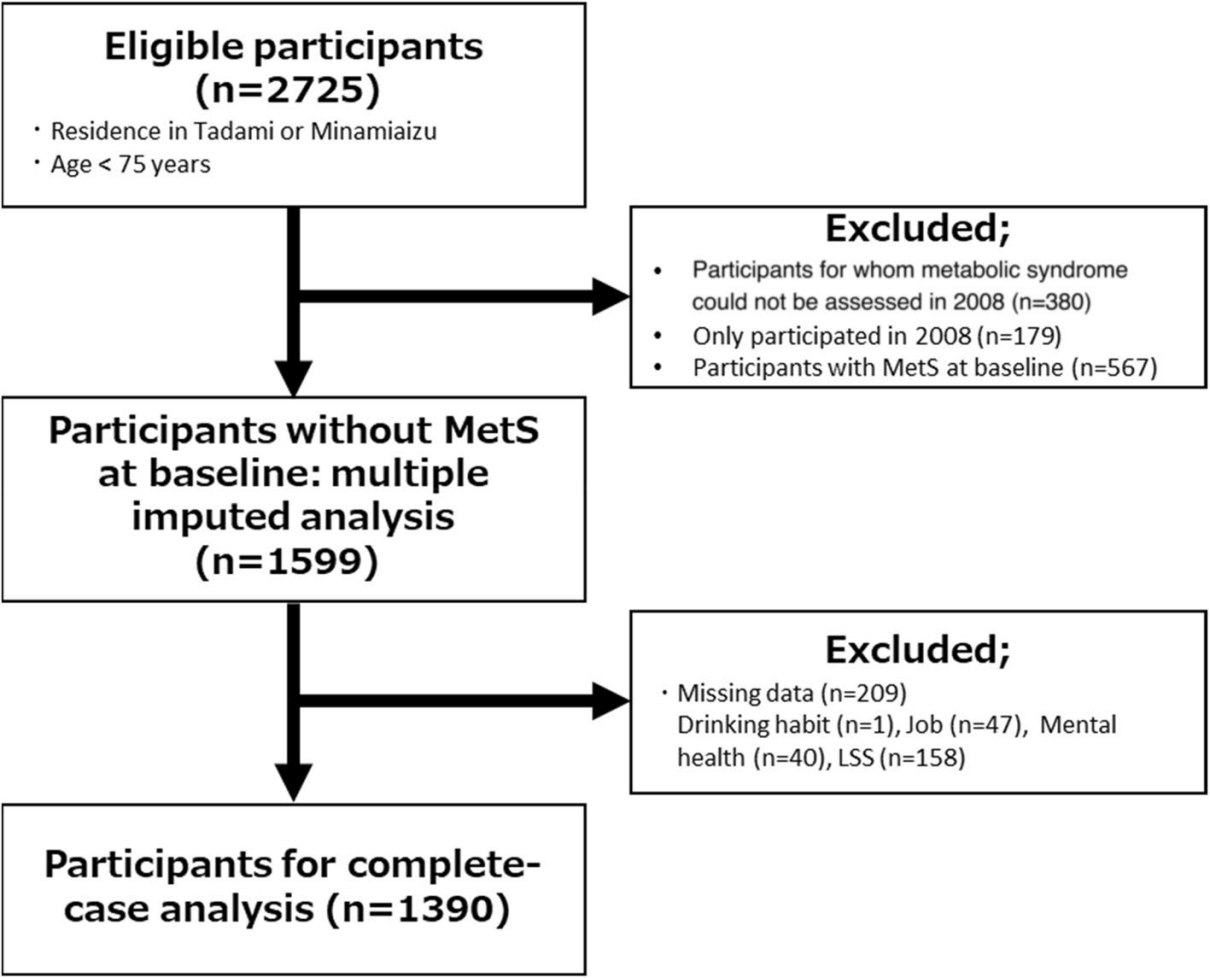
Flowchart for the study. A total of 1390 subjects were included in the complete-case analysis, and 1599 in the multiple imputation sensitivity analysis. *MetS* metabolic syndrome, *LSS* lumbar spinal stenosis.

A flowchart of the analyzed population is shown in Fig. 1. Of the 2725 participants, 1126 were excluded, leaving 1599 subjects for this study. Of these, 1390 participants were analyzed as complete cases having no missing data. The characteristics of the study population compared based on the presence of lumbar spinal stenosis are summarized in Table 1. The mean (SD) age was 62.3 (9.0) years, and 734/1390 (52.8%) participants were females. Lumbar spinal stenosis was more prevalent in subjects with enlarged waist circumference and obesity at baseline (Table 2).

**Table 1:** Characteristics of participants according to lumber spinal stenosis (*n* = 1390).

Variable	LSS		*P*-value
	− (*n* = 1309)	+ (*n* = 81)	
Age, mean (SD), years	62.1 (9.1)	65.3 (7.0)	0.002
**Sex, *n* (%)**			0.96
Female	691 (52.8)	43 (53.1)	
BMI, mean (SD), kg/m^2^	23.1 (2.8)	24.5 (3.0)	< 0.001
**Smoking status, *n* (%)**			0.91
Current	220 (16.8)	14 (17.3)	
**Drinking habits, *n* (%)**			0.54
Never	419 (32.0)	26 (32.1)	
Rarely	271 (20.7)	13 (16.1)	
Sometime	284 (21.7)	16 (19.8)	
Every day	335 (25.6)	26 (32.1)	
**Job, *n* (%)**			0.04
Yes	716 (54.7)	54 (66.7)	
Mental health, mean (SD)	52.2 (9.8)	49.4 (10.1)	0.01
**Physical activity, *n* (%)**			0.45
Low	326 (24.9)	21 (25.9)	
Moderate	270 (20.6)	21 (25.9)	
High	713 (54.5)	39 (48.1)	
**Constituent factors of MetS, mean (SD)**			
Waist circumference, cm	83.4 (7.7)	86.5 (7.9)	< 0.001
Triglyceride, mg/dL	93.4 (77.9)	90.7 (33.1)	0.75
High-density lipoprotein cholesterol, mg/dL	64.6 (14.2)	64.3 (15.2)	0.83
Systolic blood pressure, mmHg	130.7 (16.2)	135.7 (16.4)	0.009
Diastolic blood pressure, mmHg	78.2 (9.7)	79.0 (9.1)	0.47
HbA1c, %	5.0 (0.5)	5.0 (0.4)	0.67
**MetS, *n* (%)**			0.03
Yes	485 (37.1)	40 (49.4)	

*MetS* metabolic syndrome, *BMI* body mass index, *LSS* lumbar spinal stenosis, *HbA1c* hemoglobin A1c.

**Table 2:** Relationship between lumber spinal stenosis and the metabolic syndrome components and body mass index at baseline (*n* = 1390).

Variables at baseline, *n* (%)	LSS		*P*-value
	− (*n* = 1309)	+ (*n* = 81)	
Enlarged waist circumference	543 (41.5)	43 (53.1)	0.04
BMI ≧ 25 kg/m^2^	301 (23.0)	37 (45.7)	< 0.001
Elevated triglyceride level	96 (7.3)	2 (2.5)	0.10
Reduced high-density lipoprotein cholesterol	35 (2.7)	3 (3.7)	0.58
Elevated blood pressure	786 (60.1)	56 (69.1)	0.10
Elevated glucose	218 (16.7)	15 (18.5)	0.66
**Number of MetS components at baseline**			0.07
0	199 (15.2)	7 (8.6)	
1	542 (41.4)	29 (35.8)	
2	568 (43.4)	45 (55.6)	

Enlarged waist circumference: ≥ 80 cm in females or ≥ 90 cm in males; elevated triglyceride level: ≥ 150 mg/dL, or taking a drug prescribed for high triglycerides; low high-density lipoprotein cholesterol: < 50 mg/dL in females or < 40 mg/dL in males, or taking drugs prescribed for low high-density lipoprotein cholesterol; elevated blood pressure: systolic blood pressure ≥ 130 mmHg and/or diastolic blood pressure ≥ 85 mmHg, or being under antihypertensive drug treatment; elevated glucose: hemoglobin A1c ≥ 5.6% or taking a drug prescribed for elevated glucose.

*BMI* body mass index, *LSS* lumbar spinal stenosis, *MetS* metabolic syndrome.

### Metabolic syndrome incidence in the overall case analysis

The cumulative incidence of metabolic syndrome during the 3.89 (1.96) years follow-up period was 525 (37.8%). The relationship between lumbar spinal stenosis and metabolic syndrome incidence is shown in Table 3. The presence of lumbar spinal stenosis in participants without metabolic syndrome at baseline was associated with the development of metabolic syndrome in the univariable Cox regression model (HR, 1.48; 95% CI 1.08–2.05). The association between the presence of lumbar spinal stenosis and metabolic syndrome development persisted even after adjusting for possible confounders (HR, 1.41; 95% CI 1.02–1.95). The presence of lumbar spinal stenosis in participants without metabolic syndrome at baseline in the group aged < 65 years was associated with the development of metabolic syndrome in the multivariable Cox regression model (HR, 2.28; 95% CI 1.38–3.75; Supplementary Table S1), while participants aged ≥ 65 years showed no association (HR, 1.06; 95% CI 0.69–1.63; Supplementary Table S2).

**Table 3:** Relationship between lumber spinal stenosis and metabolic syndrome incidence using Cox regression analysis in complete cases (*n* = 1390).

Variable	Univariable model		Multivariable model	
	HR	95% CI	HR	95% CI
**LSS**				
−	Ref		Ref	
+	1.48	1.08–2.05	1.41	1.02–1.95
Age	1.03	1.01–1.04	1.01	1.00–1.02
**Sex**				
Male	Ref		Ref	
Female	1.39	1.16–1.65	1.01	0.82–1.25
**Smoking status**				
Never/former	Ref		Ref	
Current	0.70	0.55–0.91	1.07	0.81–1.41
**Drinking habits**				
Never	Ref		Ref	
Rarely	1.35	1.05–1.74	1.37	1.05–1.80
Sometimes	1.60	1.26–2.04	1.62	1.24–2.13
Everyday	1.56	1.23–1.96	1.48	1.14–1.92
**Number of MetS components at baseline**				
0	Ref		Ref	
1	2.90	1.86–4.53	2.95	1.88–4.63
2	7.44	4.83–11.46	7.17	4.62–11.14
Mental health	1.01	1.00–1.02	1.01	1.00–1.02

*MetS* metabolic syndrome, *LSS* lumbar spinal stenosis, *HR* hazard ratio.

### Incidence of metabolic syndrome components and obesity in the overall case analysis

Of the five metabolic syndrome components and obesity, the risk for new occurrence of enlarged waist circumference and elevated triglyceride during follow-up was higher in patients with lumbar spinal stenosis at baseline than in those without (adjusted HR, 1.93; 95% CI 1.06–3.49 and adjusted HR, 1.49; 95% CI 1.04–2.13, respectively; Table 4).

**Table 4:** Relationship between lumber spinal stenosis and the incidence of the metabolic syndrome components and body mass index in participants without each component at baseline.

Outcome variable	Cumulative incidence	Univariable model		Multivariable model	
	*n* (%)	HR	95% CI	HR	95% CI
**Enlarged waist circumference (*n* = 804)**					
LSS−	147 (19.19)	Ref		Ref	
LSS+	12 (31.58)	1.78	0.99–3.21	1.93	1.06–3.49
**BMI ≧ 25 kg/m**^**2**^** (*n* = 1052)**					
LSS−	107 (10.62)	Ref		Ref	
LSS+	5 (11.36)	1.20	0.49–2.93	1.27	0.51–3.14
**Elevated triglyceride (*n* = 1292)**					
LSS−	344 (28.36)	Ref		Ref	
LSS+	33 (41.77)	1.65	1.16–2.36	1.49	1.04–2.13
**Reduced high-density lipoprotein cholesterol (*n* = 1352)**					
LSS−	97 (7.61)	Ref		Ref	
LSS+	1 (1.28)	0.19	0.03–1.34	0.16	0.02–1.16
**Elevated blood pressure (*n* = 548)**					
LSS−	297 (56.79)	Ref		Ref	
LSS+	17 (68.00)	1.38	0.85–2.25	1.23	0.75–2.02
**Elevated glucose (*n* = 1157)**					
LSS−	100 (9.17)	Ref		Ref	
LSS+	7 (10.61)	1.26	0.58–2.70	1.18	0.54–2.55

The primary explanatory variables in all models was lumbar spinal stenosis.

Each model was analyzed without the prevalence of each component. Cox regression analysis was used to complete missing data.

All models were adjusted for age, sex, smoking status, alcohol consumption, number of metabolic syndrome components at baseline and mental health.

Enlarged waist circumference: ≥ 80 cm in females or ≥ 90 cm in males; elevated triglyceride level: ≥ 150 mg/dL, or taking a drug prescribed for high triglycerides; low high-density lipoprotein cholesterol: < 50 mg/dL in females or < 40 mg/dL in males, or taking drugs prescribed for low high-density lipoprotein cholesterol; elevated blood pressure: systolic blood pressure ≥ 130 mmHg and/or diastolic blood pressure ≥ 85 mmHg, or being under antihypertensive drug treatment; elevated glucose: hemoglobin A1c ≥ 5.6% or taking a drug prescribed for elevated glucose.

*BMI* body mass index, *LSS* lumbar spinal stenosis, *HR* hazard ratio.

### Incidence of metabolic syndrome in the multiple imputations analysis

The results from multiple imputations and complete cases showed similar trends. The presence of lumbar spinal stenosis in participants without metabolic syndrome at baseline influenced the incidence of metabolic syndrome in the univariable and multivariable Cox regression models (univariable model: HR, 1.51; 95% CI 1.11–2.06; multivariable model: HR, 1.47; 95% CI 1.08–2.00; Supplementary Table S3).

## Discussion

We prospectively investigated the association between lumbar spinal stenosis and the incidence of metabolic syndrome in community-dwelling adults. Our study identified lumbar spinal stenosis as a risk factor for metabolic syndrome incidence.

To our knowledge, this was the first study to prospectively analyze the relationship between lumbar spinal stenosis and metabolic syndrome in a community setting. Additionally, we determined that triglyceride levels, a component of metabolic syndrome, was associated with lumbar spinal stenosis. Our results also revealed a similar trend for enlarged waist circumstance. Previous cross-sectional studies have shown an association between lumbar spinal stenosis and lifestyle-related diseases, although the definitions differed between studies. Lotan et al.^22^ investigated 537 patients with spinal stenosis in a hospital setting. Hypertension (23.2 vs. 7.8%) and diabetes mellitus (13.6 vs. 5.9%) were more prevalent in the study group than the general population. In another study of 395 patients diagnosed with spinal stenosis, degenerative disk disease, or osteoporotic vertebral fractures, patients in the spinal stenosis group had a significantly higher rate of diabetes mellitus than the other groups^23^. Uesugi et al.^11^ investigated the relationship between lumbar spinal stenosis and comorbidities in 526 patients with lumbar spinal stenosis in a community setting. The results showed significant associations between diabetes mellitus and high blood pressure and lumbar spinal stenosis. Maeda et al.^8^ also showed that diabetes mellitus and low ankle-brachial index values were significantly associated with lumbar spinal stenosis. Our results differed from those of previous studies, all of which were cross-sectional. Lower cumulative incidence during study periods may be affected because of the longitudinal study design. The commutative incidence of BMI (112/1052; 10.6%), lower high-density lipoprotein cholesterol levels (98/1352; 7.2%), and elevated glucose levels (107/1157; 9.2%) was lower than that of waist circumference (159/804; 19.5%) or triglyceride levels (377/1292; 29.2%) (Table 4). Our results provide further understanding regarding these relationships, although additional research is warranted.

Our study showed that the presence of lumbar spinal stenosis was associated with the incidence of metabolic syndrome in only participants aged < 65 years. Previous Asian-based studies^24,25^ have reported that the prevalence of metabolic syndrome peaks at the age of 60–70 years, while the number of geriatric syndromes, such as delirium, falls, incontinence, and frailty, increases in those aged ≥ 65 years^26^. This suggests that there may be a diversity of factors associated with metabolic syndrome in older age. The effect of lumbar spinal stenosis on metabolic syndrome may be reduced in individuals aged ≥ 65 years.

As an etiological explanation for our results, low physical activity might mediate the association between lumbar spinal stenosis and metabolic syndrome. Norden et al.^12^ investigated physical activity using an accelerometer in 75 patients with lumbar spinal stenosis. Most participants were extremely sedentary and did not meet the guidelines for physical activity. Patients with degenerative musculoskeletal disorders are limited in their walking ability^27^, and physical activity is decreased in community-dwelling older individuals with musculoskeletal pain^28^. It is known that low levels of physical activity and sedentary behavior lead to lifestyle-related diseases in middle-aged and older people^29,30^. Several studies showed reverse causality between lumbar spinal stenosis and/or lower back pain and lifestyle-related diseases. One review showed that as arteriosclerosis due to hypertension and dyslipidemia progresses, posterior aortic wall calcification occurs, leading to the progression of intervertebral disc degeneration^31^. Other studies found that diabetes mellitus leads to pathological changes and early joint degeneration^32,33^. Research on the relationship between lumbar spinal stenosis and lifestyle-related diseases searches for causation; however, further research is needed to find whether direct, indirect, or lifestyle factors exist.

Although the etiological relationship between lumbar spinal stenosis and metabolic syndrome has not been clarified, this study aimed to investigate one aspect of the relationship between them in a long-term community-dwelling cohort. Despite the important findings of this study, several limitations should be considered. First, there might be information bias in this study. We used a questionnaire, a non-objective assessment tool, to identify lumbar spinal stenosis. The sensitivity and specificity of this questionnaire were 84% and 78%, respectively^16^. Some of the participants reporting having lumbar spinal stenosis may not have actually been diagnosed with lumbar spinal stenosis. We think, however, that using this questionnaire was the best way to test our hypothesis in a community setting. The definition of metabolic syndrome has been modified and changed over time. Although we used the most recent definition available at the time of the study, these relationships could become unstable in the future. Second, we had no data on treatments for lumbar spinal stenosis and did not follow-up on lumbar spinal stenosis symptoms. Some of the participants identified as having lumbar spinal stenosis might have sought care and treatment for the condition. Since we hypothesized that lumbar spinal stenosis at baseline was associated with the incidence of metabolic syndrome, improvements in spinal symptoms may have protected them from the incidence of metabolic syndrome. The results of this study are not overestimated. Third, we stratified follow-up by years, not months and/or days. As special health checkups or health checkups were held frequently, the impact of time was small. Fourth, as this was an observational study; therefore, there might be other potential biases between lumbar spinal stenosis and metabolic syndrome. Finally, this study was conducted in a small area in Japan. Therefore, the generalizability of our results should be carefully considered. Further studies are required to validate and generalize the results of this study.

## Conclusion

We prospectively investigated the association between lumbar spinal stenosis and metabolic syndrome incidence. Lumbar spinal stenosis was identified as a risk factor for metabolic syndrome occurrence. By maintaining an appropriate physical activity level and moderate exercise, it is possible to prevent the occurrence of metabolic syndrome. The study findings provide useful information for the prevention and management of the metabolic syndrome. We recommend conducting an etiological study that would include a comparison using objective measures in lumbar spinal stenosis and metabolic syndrome or a study to determine how decreasing physical activity due to lumbar spinal stenosis mediates the incidence of metabolic syndrome.

## Supplementary Information

Supplementary Tables.

## Data Availability

The datasets generated during and/or analysed during the current study are not publicly available due to ethical restrictions, however further analyses may be completed by the authors on reasonable request.
