# Supplementary material for: Impact of lumbar spinal stenosis on metabolic syndrome incidence in community-dwelling adults in Aizu cohort study (LOHAS)
Source: Sci Rep. 2022 Jul 4;12:11246. doi: 10.1038/s41598-022-15173-y (PMC9253139; doi:10.1038/s41598-022-15173-y)
Supplement: Supplementary file 1 — Supplementary Tables. [file 41598_2022_15173_MOESM1_ESM.docx]

**Supplementary Information**

**Impact of lumbar spinal stenosis on metabolic syndrome incidence in community-dwelling adults in Aizu cohort study (LOHAS)**

Rei Ono PhD ^1, 2) *^, Misa Takegami PhD ^3)^, Yosuke Yamamoto PhD ^4)^, Shin Yamazaki PhD ^5)^, Koji Otani DMSc ^6)^, Miho Sekiguchi PhD ^6)^, Shin-Ichi Konno PhD ^6)^, Shin-Ichi Kikuchi PhD ^6)^, Shunichi Fukuhara DMSc ^7, 8)^

**Supplementary Table S1. Relationship between lumber spinal stenosis and metabolic syndrome incidence using Cox regression analysis in participants aged <65 years (n = 678)**

| **Variable** | **Univariable model** | |  | **Multivariable model** | |
| --- | --- | --- | --- | --- | --- |
|  | **HR** | **95% CI** |  | **HR** | **95% CI** |
| **LSS** |  |  |  |  |  |
| **-** | **ref** |  |  | **ref** |  |
| **+** | **2.42** | **1.48–3.96** |  | **2.28** | **1.38–3.75** |

**LSS, lumbar spinal stenosis; HR, hazard ratio.**

**All models were adjusted for age, sex, smoking status, alcohol consumption, number of metabolic syndrome components at baseline, and mental health**

**Supplementary Table S2. Relationship between lumber spinal stenosis and metabolic syndrome incidence using Cox regression analysis in participants aged ≥65 years (n = 712)**

| **Variable** | **Univariable model** | |  | **Multivariable model** | |
| --- | --- | --- | --- | --- | --- |
|  | **HR** | **95% CI** |  | **HR** | **95% CI** |
| **LSS** |  |  |  |  |  |
| **-** | **ref** |  |  | **ref** |  |
| **+** | **1.07** | **0.70–1.63** |  | **1.06** | **0.69–1.63** |

**LSS, lumbar spinal stenosis; HR, hazard ratio.**

**All models were adjusted for age, sex, smoking status, alcohol consumption, number of metabolic syndrome components at baseline, and mental health**

**Supplementary Table S3. Relationship between lumbar spinal stenosis and metabolic syndrome incidence using Cox regression analysis in multiple imputation (*n* = 1599)**

| **Variable** | **Univariable model** | |  | **Multivariable model** | |
| --- | --- | --- | --- | --- | --- |
|  | **HR** | **95% CI** |  | **HR** | **95% CI** |
| LSS |  |  |  |  |  |
| - | ref |  |  | ref |  |
| + | 1.51 | 1.11–2.06 |  | 1.47 | 1.08–2.00 |
| Age | 1.02 | 1.01–1.03 |  | 1.01 | 1.00–1.02 |
| Sex |  |  |  |  |  |
| Male | ref |  |  | ref |  |
| Female | 1.36 | 1.16–1.60 |  | 0.99 | 0.82–1.20 |
| Smoking status |  |  |  |  |  |
| Never | ref |  |  | ref |  |
| Current | 0.66 | 0.52–0.84 |  | 0.97 | 0.75–1.26 |
| Drinking habits |  |  |  |  |  |
| Never | ref |  |  | ref |  |
| Rarely | 1.36 | 1.08–1.72 |  | 1.34 | 1.06–1.74 |
| Sometimes | 1.55 | 1.24–1.93 |  | 1.54 | 1.20–1.98 |
| Everyday | 1.51 | 1.22–1.87 |  | 1.40 | 1.10–1.77 |
| Number of MetS components at baseline |  |  |  |  |  |
| 0 | ref |  |  | ref |  |
| 1 | 2.54 | 1.71–3.78 |  | 2.53 | 1.69–3.78 |
| 2 | 6.79 | 4.63–9.97 |  | 6.44 | 4.35–9.53 |
| Mental health | 1.01 | 1.00–1.01 |  | 1.00 | 1.00–1.02 |

MetS, metabolic syndrome; LSS, lumbar spinal stenosis; HR, hazard ratio.
